# The social facilitation of eating or the facilitation of social eating?

**DOI:** 10.1186/s40337-017-0146-2

**Published:** 2017-04-27

**Authors:** C. Peter Herman

**Affiliations:** University of Toronto, Toronto, Canada

**Keywords:** Food intake, Social facilitation, Overindulgence

## Abstract

People eat more when they eat in groups. Various explanations have been offered for this “social facilitation” of eating. We consider these explanations and find most of them wanting, especially insofar as they do not take into account the increased per capita provision of food when people eat together. We suggest that people often prefer to eat in groups precisely because it offers them an opportunity to overindulge.

## Plain English summary

People eat more when they eat in groups. Why? It turns out that when people eat in groups, more food is available to each of them, on average. We propose that people arrange for more food to be available when they plan to eat in a group because eating in a group - at least a group of friends - makes it possible for people to eat more than they ordinarily would. Such overindulgence is one of the pleasures of group eating.

The social facilitation of eating refers to the empirical fact that people eating with others eat more - often a lot more - than do people eating alone. The basic phenomenon contrasts solo diners with group diners, with the groups consisting of 2 or more people [1]. The effect seems to follow social impact theory [2], such that the more people there are in the group, the bigger the effect, although the curve is negatively accelerated [3].

## Background

Before proceeding further, it is worth considering some intriguing questions about what constitutes a group. If you go to the cinema alone, and are sitting by yourself along with scores of strangers, are you alone or in a group? Hirsch and Kramer [4], in a military canteen setting, found that people ate more when they ate socially, in a group. These authors defined non-social eating as “eating alone or as part of an undifferentiated large group of 50 to 70 people.” Thus, eating in a crowd (as opposed to eating in a group) does not facilitate intake. This study raises (but does not answer) fascinating questions about what exactly constitutes the sort of group that facilitates eating.

To return to the basic social facilitation effect, the discovery of what he called the “social correlation” - the more people in the group, the greater the intake for each individual in the group, on average - is usually associated with the name John de Castro, who, starting in the late 1980s [5], conducted several diary studies. People kept track of when and how much they ate, and the circumstances surrounding each eating episode. People clearly reported eating more when they ate with others. This effect was demonstrated to be independent of a variety of possible confounds. For instance, it might be that people tend to eat more at dinner than at breakfast, and are more likely to eat with other people at dinner than at breakfast. However, the social facilitation effect was found at breakfast, lunch, and dinner, separately. Parallel potential confounds were eliminated in the diary studies: people ate more in groups on weekdays and weekends [6], at meals with and without alcohol, and at meals eaten at home or at restaurants [7]. de Castro concluded that eating with others has a larger impact on the amount eaten than almost any other factor that we know of [8]. (Of course, we often forget that almost all meals consist of food that the eater finds palatable, and if we were to substitute unpalatable food, intake would drop off precipitously, as a main effect; so it may be that palatability has a stronger main effect than does social facilitation, but a full range of palatability is hardly ever considered.)

de Castro’s diary studies have been supported, more or less, by several laboratory studies, a few of which predated de Castro’s work (but which did not use the term social facilitation) (e.g., [9]).

## Main text

Explanations for the social facilitation of eating are surprisingly inconclusive. de Castro [8, 10] favored a “time extension” theory of social facilitation. He noted that the meal takes longer when people eat in groups, and he concluded that this extra time led to extra eating. Although de Castro did not say so directly, his hypothesis implied that exposure to food cues drives eating, in what amounts to a simple stimulus-response arc. The greater the exposure to food cues, the more one will eat, so a longer time at a food-laden table results in greater intake. Pliner, Bell, Hirsch, and Kinchla [11] independently manipulated group size and meal duration and found that it was meal duration, not group size, that predicts amount eaten, supporting the cue-exposure hypothesis. In the real world, however, group size and meal duration are strongly confounded, at least when the group consists of friends. We may ask *why* groups take longer to eat. One easy answer is that groups engage in much more socializing, but does that answer really explain things? If groups spend more time talking amongst themselves, how does that result in greater food intake? If you partition the episode into eating time and talking time, then adding talking does not necessarily add eating. Maybe if there are three people in your group, you do not eat more while you are talking, but you do eat more when the others are talking. More people means more conversation, including more opportunity to eat while listening. This issue has never been examined carefully; and as we shall see, there is reason to think that there is something else going on here.

Consider some of the other mechanistic explanations for social facilitation that have been proposed. One is that the presence of other people induces arousal, and arousal may increase hunger, or increase dominant responses, as Zajonc [12] argued earlier in an analysis of social facilitation that preceded studies of socially facilitated food intake in humans. If the dominant response in the presence of food is to eat, then arousal may potentiate this response. Arousal could conceivably activate appetite, although it could also suppress appetite; some people eat more when they are stressed and other people eat less [13]. Some rats freeze when they’re stressed, but a tail-pinch stressor can make them eat more [14]. The effects of arousal or emotional agitation on food intake are complicated; and in any case, we shall conclude that a simple arousal model cannot account for the data.

Another candidate is distraction. The distraction explanation hinges on the notion that people will eat freely in the presence of palatable food, stopping only when they become sated or when some other inhibitory factor intervenes [15]. One inhibitory factor might be an acute awareness of how much one is eating, perhaps verging on the inappropriate, or of how full one is feeling [16]. Eating in a group might distract you from your customary attention to how much you are eating or how full you feel. The result could be overeating, often without awareness (just as watching TV while eating can result in overeating, again without awareness) [17]. These types of explanations are premised on the notion that these factors simply make one eat more (in the case of arousal) or interfere with normal constraints on eating (in the case of distraction).

Another possibility is modeling. Modeling studies usually involve a confederate who eats a lot or a little, with the naïve participant following suit [18]. In the social facilitation paradigm, there is no explicit model; everyone is a naïve participant; but it remains possible that you will perceive the others as eating a lot, or at least more than you yourself are eating, which leads you to eat more so as to keep up. There are different possibilities for why people keep up with others’ intake. We may want to maximize our intake [19]; so if we see others eating more, or convince ourselves that they are eating more, then we feel entitled to increase our own intake correspondingly. Or perhaps one keeps up with the others not because one wants to but because one believes that one has to. Matching other people’s intake is a way of establishing a connection with them, of fulfilling an implicit social contract, and of not embarrassing them by eating less than they do [20]. (People resent it when you eat less than they do [21].) There are no useful data on how much people in the social facilitation situation perceive their eating companions to be eating, but it appears that people are eager to maximize their eating as long as the food is palatable. Food intake is limited by inhibitory factors, including norms set by one’s eating companions; so if your eating companions are eating more, you are in effect entitled to eat more.

One important aspect of social facilitation effect that has not been mentioned so far is that it is confined to friends and family. People do not eat more when the group consists of strangers [1]. With strangers, one supposes, impression-management concerns become magnified. We try harder to impress strangers than friends [22] - friends can almost be defined as people who accept you to the point where you do not have to impress them - and one way to impress people - in this case, strangers - is to eat sparingly. Excessive eating attracts negative evaluations. People who eat a lot are perceived as lacking in various positive attributes, including attractiveness, intelligence, and self-control. The only positive attribute associated with eating a lot is “fun-loving” [23].

Another, even more important aspect of social facilitation that we have not yet considered is how much food is available to the social group. In the laboratory, the amount of food available to research participants is effectively unlimited. The researchers do not want food intake to be artificially constrained by participants eating all of it and being forced to stop. In the real world, however, unlike the laboratory, people have to supply or acquire their own food, and they cannot or will not arrange for unlimited food. Also, it is clear from empirical studies that people tend to eat most or all of the food that they serve themselves. Brunstrom [24] has documented the fact that people judge how much food they will need, and after experience with different foods, people usually become fairly accurate.

So when we observe that people (at least friends) eat more in groups, it is not a matter of solo diners and group diners starting out with the same amount of food on their plates and that solo diners leave more of their food uneaten whereas group diners clean their plates. In the real world, solo diners and group diners both eat most of whatever food they have provided for themselves [25]. What this means, and what is often overlooked in discussions of social facilitation, is that people eating in groups start out with more food (per capita); they provide themselves with more food before the meal even begins. Cavazza et al. [25] found, in an Italian restaurant - a real Italian restaurant, in Italy - that the number of dishes ordered was a direct function of group size, and this function applied per capita; that is, as the size of the group increased, each diner was allotted more food. Furthermore, the amount of leftovers was unrelated to group size. People who ate in large groups ate almost all of their allotted food, and solo diners ate almost all of their allotted food, even though the group diners each started off with more food. What we are seeing, then, is not simply that people in groups eat more, but that they start off with more, in what might be called the social precilitaiton of eating [1].

So now we have arrived at the point where we have to ask ourselves why the group provides itself with more food (per capita) than does the individual. (Of course, it is not necessarily the case that the group provides itself with the meal. Often the meal is organized by a smaller subset of the group, or possibly just a single member of the group, or even someone who is not part of the group at all but who is aware that a group will be dining together. In all of these cases, whoever organizes the meal tends to provide more food per capita as the size of the group increases. Often this means adding courses or dishes “for the table” as group size increases.)

People enjoy company when they eat (and even when they’re not eating). This preference is not absolute. Sometimes we like our privacy, and Pliner and Bell [26] cite some celebrities who argue for the social and gustatory benefits of eating alone; but this is a minority opinion. There is considerable research evidence that people do not like to eat alone, especially in public [27]. Eating alone can make you feel lonely; and if you are eating alone at a restaurant, you may worry that other people will regard you negatively, as a social loser [28]. It is even conceivable that when people eat alone, they are uncomfortable because do not know how much to eat (whereas eating companions provide them with some clues). So there is some benefit to eating in the company of friends, above and beyond the pleasure of the companionable interaction itself. Still, that does not explain why people eat more when they eat with friends.

One of the things that people enjoy about eating with friends is the company of the friends, but another thing that they enjoy is the eating itself. As we have suggested earlier, people want to maximize their intake of palatable food. Eating is a sensual experience and if not for the societal proscriptions against eating various indulgent foods or eating too much overall, we would be happy to indulge freely. Eating with friends promotes such freedom and allows people to push the boundaries of acceptable intake. (Of course, this does not happen when we eat with strangers, whom we are trying to impress, or with false friends who are actually not on our side but rather watching us carefully to catch us out.) We have already discussed the possibility that people overestimate how much their dining companions are eating, which in turn allows them to eat more without incurring the negative imputations associated with overeating. So maybe in the group, everyone can overeat a little while convincing themselves that they are all eating appropriately. Another possibility is that people will overeat, knowing full well that they are overeating, on the condition that everyone else does so too. In effect, when a group of friends gets together to enjoy a meal (and each other’s company), there may be an implicit or even an explicit agreement that overindulgence will be condoned or even encouraged. If only one person will summon up the courage to order dessert, the rest will all happily fall in line. This complicit overindulgence may be especially attractive to dieters, who are often looking for an excuse to overeat; but it is attractive to everyone. Eating in groups with friends is construed as a special occasion, during which overindulgence is permitted.

From a public-health perspective, the problem is that eating in groups with friends has become so commonplace that what used to be a special occasion is now a regular occurrence, with a corresponding increase in the frequency of overindulgence. And overindulgence, as we know, is a threat to the well-being of the individual and society - again, from a public-health perspective. From a social and gustatory perspective, however, these occasions, if no longer exactly special, are nevertheless pleasant, public health considerations notwithstanding.

## Conclusions

It is customary to bemoan the increasing frequency of socially facilitated eating. Not only are people eating with friends more often, but those meals are not particularly diet-friendly. When people overeat in groups, they tend to do so on foods other than salad. One conclusion is that people should eat alone as often as possible. (Sometimes dine-alone crusaders make an exception for the elderly, who tend to eat too little, and who might benefit from the increased indulgence that happens in groups [29]. It could likewise be argued that anorexia nervosa patients might benefit from social eating, but it is difficult to imagine a group of such patients jointly deciding to overcome their inhibitions; the intense concerns about overindulgence that are at the heart of the disorder seem unlikely to dissolve in a group setting.) The recommendation that you should eat alone so as to avoid overindulgence, however, is not the sort of advice that people are likely to listen to. People want the experience of caloric overindulgence in the pleasant company of their friends, friends who are often actively encouraging such overindulgence.

If people simply wanted to socialize together, there would be no need to provide extra food. The fact that social eating tends to involve the provision of extra food indicates that indulging in more food - making the meal into something of a feast - is an essential part of social eating. We eat together because we want to socialize; but part of the reason that we want to socialize is so that we can eat more.

But why do we not eat indulgently when we eat alone? It seems likely that people who eat alone are concerned about overeating. We have argued that eating with strangers highlights this concern, but this concern may well apply to the solo diner, who is not worried what others will think of her but rather what she will think of herself if she overeats. In the absence of indulgent dining companions, the solo dinner has to play it safe. When there are others around to share the guilt - or more accurately, to help dismiss the guilt - we can safely overindulge.

Amongst friends, we can let our guard down and enjoy ourselves. Remember that “fun loving” is one of the few positive attributes associated with eating a lot. Bringing that fun-loving, indulgent eating style to a social occasion makes it all the better.
